# Supplementary material for: Clinical characteristics and outcomes of critically ill patients with COVID-19 admitted to an intensive care unit in London: A prospective observational cohort study
Source: PLoS One. 2020 Dec 15;15(12):e0243710. doi: 10.1371/journal.pone.0243710 (PMC7737956; doi:10.1371/journal.pone.0243710)
Supplement: Attachment — Submitted filename: Response to Reviewers.docx [file pone.0243710.s001.docx]

London, United Kingdom

Sunday, 22^nd^ November 2020

Dear Dr Lazzeri,

Many thanks for considering our manuscript titled “Clinical characteristics and outcomes of critically ill patients with COVID-19 admitted to an intensive care unit in London: a prospective observational cohort study”. We are grateful for your comments and those of the peer reviewers. We have prepared a revised manuscript and a point-by-point response to your comments and those of the peer reviewer.

**Responses to Academic Editor**

1. Please ensure that your manuscript meets PLOS ONE's style requirements, including those for file naming.

We have actioned this point in the revised submission.

2. Please clarify the name of the IRB that classified the study as a non-interventional service evaluation and waived participant consent.

The legal framework governing medical research in the United Kingdom does not require institutional review board or ethics committee approval for studies that meet the definition of non-interventional service evaluations, nor does it require an IRB to decide that a study meets this definition. The study was registered with the Institutional Research Office of the Royal Free London NHS Foundation Trust as a non-interventional service evaluation. Even if this were not the case, and participant consent would ordinarily be required, the UK government has instituted a consent waiver for the purposes of COVID-19 research (notice under regulation 3(4) of the Health Service (Control of Patient Information) Regulations 2002).

3. Please see our revised competing interests statement below:

   I have read the journal's policy and the authors of this manuscript have the following competing interests:

DM has received consultancy and lecture fees from Edwards Lifesciences and Siemens Healthineers; none of the other authors have any conflicts of interest to report. This does not alter our adherence to PLOS ONE policies on sharing data and materials.

4. Data availability

   This study utilised data collected from patients admitted to hospital with COVID-19. This confidential patient data is protected by ethical considerations (patients have a right to confidentiality), the professional obligations of the researchers (who are bound by the policies of the General Medical Council, the statutory body that regulates doctors in the UK) and by UK law. Unrestricted sharing of the data underpinning the study is therefore not possible on ethical and legal grounds. We do not believe it is possible to sufficiently anonymise the data while maintaining its usefulness for research. The dataset is extremely granular and concerns a relatively small number of individuals. We are therefore concerned that individuals would be potentially identifiable even if overt identifiers (name, date of birth, etc.) were removed. Furthermore, repurposing the data so that it could be shared would not be compatible with the legal basis (service evaluation) under which we have conducted our study.

We would be happy to share the data underpinning the study to facilitate replication of our findings or further research, but this needs to be done in a manner compatible with UK law. This would involve a group of researchers obtaining ethical and research governance approvals and their institution entering into a data sharing agreement with ours. We have provided contact details for our Institutional Research Office in our data availability statement.

We have updated our data sharing agreement:

The data that support the findings of this study are not publicly available out of consideration for patient confidentiality. On account of the highly granular nature of the clinical dataset it is not possible to anonymise it sufficiently to prevent reidentification of participants without jeopardising its usefulness for research. The Institutional Research Office (rf.randd@nhs.net) can facilitate access to the data for appropriately qualified individuals provided that the correct ethical permissions and data sharing agreements are in place.

**Responses to Reviewer**

Thank you for your detailed and careful consideration of our manuscript.

1. Inclusion bias

   We entirely agree with the reviewer’s comments regarding inclusion bias. The subset of patients admitted to ICU represents a group who were critically unwell with COVID-19, and who were thought likely to benefit from admission to critical care.

As an organisation we devised a guideline on the care of patients with COVID-19. This was developed jointly by physicians from the ICU, acute medicine and the emergency department. We employed a number of objective criteria to assess illness severity (and hence need for ICU admission). We used the World Health Organisation’s definition of “critical” COVID-19 (the presence of ARDS, sepsis or septic shock) to identify those patients who definitely required admission to critical care. We used the WHO’s definition of “severe” COVID-19 (respiratory rate > 30 breaths/min; severe respiratory distress; or SpO2 < 90% on room air) to identify those at high risk of deterioration who required close monitoring. Inevitably there were patients admitted to ICU who did not meet these criteria, for example because they had comorbidities (e.g. organ transplantation) or other conditions (e.g. acute renal failure) that necessitated treatment in a critical care setting.

We used the guidance from the National Institutes of Health and Care Excellence to assess patients’ likelihood of benefitting from ICU admission. This involved calculating each patient’s Clinical Frailty Score, with the frailest patients being least likely to survive admission to critical care.

Through the use of objective criteria for illness severity requiring ICU admission, and frailty that would render ICU admission futile, we attempted to standardise decision making. The reviewer is correct to point out that an element of subjectivity is inevitable, and that thresholds for critical care admission will have changed over time and with our understanding of the disease. It is therefore likely that the mix of patients in ICU will vary between institutions, countries and even within the same institution over the stages of the pandemic.

We have updated the Methods section to explain our policy for critical care admission, and the Discussion to better explain the limitations imposed by inclusion bias. We have also placed ICU admissions in context by providing data on the total number of patients admitted to the hospital during the study period and their average age. Unfortunately our institution employs a paper-based health record and the administrative data recorded electronically is very limited, restricting our ability to provide detail of the cohort of patients admitted to hospital but not requiring ICU admission.

2. Minor: p 13 line 294 Of the 156 total admissions to ICU with COVID-19, 38 (24%) patients died on ICU, 23 (15%) patients were transferred out to other hospitals, 92 (59%) patients were discharged alive from ICU and the remaining 3 (2%) patients were still on ICU - SHOULD SAY SOMETHING LIKE "at the time of follow up "

   Thank you – we have updated the manuscript to make this clear.

We hope that we have addressed your concerns in a satisfactory fashion. Please do not hesitate to contact us should you require further information or clarification.

Yours faithfully,

**Dr Ross J Thomson BA BMBCh MRCP**
